# Impact of Spousal Addiction on Women's Marital Satisfaction and Family Peace in Turkey

**DOI:** 10.1002/brb3.71089

**Published:** 2025-11-21

**Authors:** Mehmet Ali Sen, Eda Yakit Ak, Ozden Tandogan, Ramazan Alkan, Ezgi Yarasir

**Affiliations:** ^1^ Dicle University Atatürk Health Services Vocational School Diyarbakır Turkey; ^2^ Istanbul Arel University Faculty of Health Sciences Department of Nursing Istanbul Turkey; ^3^ Department of Internal Medicine‐Psychiatry University of Health Sciences, Istanbul Basaksehı̇r Cam and Sakura City Health Practice and Research Center Istanbul Turkey; ^4^ Department of Physical Therapy and Rehabilitation Science Roy J. and Lucille A. Carver College of Medicine The University of Iowa Iowa City Iowa USA

**Keywords:** addiction, family peace, fulfillment, marriage, women

## Abstract

**Purpose:**

This study aims to investigate the impact of spousal addiction on women's marital satisfaction and family peace in Turkey.

**Methods:**

This hospital‐based case‐control study was conducted between May and September 2024 in a large city in Turkey. A total of 309 married women aged 18 years and older participated, including those married to men hospitalized for addiction and those married to non‐addicted men. Data were collected using self‐administered questionnaires covering socio‐demographic characteristics, addiction status, marital satisfaction, and the Family Peace Scale (FPS).

**Results:**

The mean age of participants was 35.57 ± 9.58 years. Women whose husbands were undergoing addiction treatment (45.72 vs. 56.70) or addicted to any substance (49.13 vs. 59.11) had significantly lower FPS scores (*p* < 0.05). Similarly, FPS scores were significantly lower for women whose husbands were addicted to cigarettes (49.61 vs. 57.17), alcohol (44.17 vs. 54.59), drugs (44.52 vs. 56.39), or medication (41.27 vs. 53.61) (*p* < 0.05). According to the results of the logistic regression analysis, a higher educational level of the woman (OR = 2.388) was associated with a reduced likelihood of the man being addicted, whereas the presence of any substance addiction in the woman (OR = 2.422), lower FPS scores (OR = 2.042), and the presence of sexual problems (OR = 1.484) were associated with an increased likelihood. (*p* < 0.05).

**Conclusion:**

This study shows that addictive behaviors harm marital satisfaction and family stability. In addition to addiction treatment, policymakers should enhance family support and preventive programs, while clinical practice should provide family therapy and communication and stress management interventions involving both individuals and their spouses.

## Graphical abstract

This study shows that addictive behaviors harm marital satisfaction and family stability. Family peace was worse for women whose husbands were addicted. Also, a higher educational level of the woman was associated with a reduced likelihood of the man being addicted, whereas the presence of any substance addiction in the woman, reduced family peace, and the presence of sexual problems were associated with an increased likelihood.

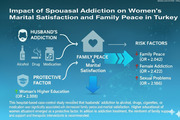

## 1 Introduction

The term “addiction” is defined as a chronic mental process in which a number of factors, including biological, psychosocial, and environmental, play a role. Those who are addicted are likely to engage in risky behaviors on a repeated basis and may require long‐term or lifelong treatment. The most prevalent addictions with adverse health consequences are tobacco, alcohol, and drug addictions. Severe addictions, such as drug or alcohol abuse, result in long‐term alterations to brain function, leading to a loss of self‐control and an inability to make sound decisions. Concurrently, the ingestion of drugs and alcohol engenders the creation of intense cravings, analogous to the physiological need for sustenance on a daily basis (OASH 2021). It is reported that 7% of the global population is affected by an alcohol use disorder, while 5.8% of the global population uses drugs. Furthermore, there has been a 23% increase in substance use over the past decade (UNODC 2023). In Turkey, the prevalence of alcohol consumption among men is 12.1%, while a report on drug users indicated that 93.8% of those engaging in substance use are male (TSI 2023; Turkish Ministry of Interior 2023).

Addiction has been linked to a range of adverse outcomes, including physical and mental health issues for individuals, as well as the deterioration of the social structure of the society in which they live and the breakdown of peace within the family. The consumption of alcohol and illicit substances can result in a loss of self‐control, heightened feelings of anger and resentment, and the emergence of conflicts within the marital relationship. Furthermore, such addictions contribute to the exacerbation of social degradation, which in turn gives rise to a culture of distrust between spouses, as well as a range of violent behaviors against women, including verbal and physical abuse and even fatalities (Abdullahi et al. 2024). The presence of alcohol and substance addictions in women's marital relationships has been demonstrated to have a generally negative effect on their levels of marital satisfaction. The actions of the addicted partner may have a detrimental impact on the emotional and psychological well‐being of the woman. Furthermore, the inability of the addicted spouse to fulfill their responsibilities may result in family unrest and conflict. This situation may result in women being unable to derive satisfaction from their marriages, which could have a detrimental impact on family harmony.

The consumption of alcohol and drugs frequently results in financial difficulties within the domestic environment, social isolation and estrangement from relatives, feelings of insecurity due to the perpetuation of unrealistic discourses, and a worsening of the problem through the denial of addiction. The use of these substances has also been linked to the development of sexual dysfunction (American Addiction Centers 2024). Alcohol and substance addictions can negatively affect women's social environments, leading to social isolation and the loss of support systems. Such circumstances reduce women's overall well‐being and threaten family stability. Therefore, it is essential to provide adequate support and protection for women coping with addicted partners, alongside strengthening public awareness and community‐based support mechanisms.

The issue of alcohol and substance addiction has been identified as a significant factor influencing the stability of marital relationships. The negative effects of addiction on family dynamics are a recurring theme in the literature (Hakim et al. 2024; Guo and Li 2022). There is evidence that women whose spouses have alcohol addiction experience more emotional and psychological difficulties, reporting lower quality of life and greater family conflict (Jones et al. 2024; Opitz et al. 2009; Sobol‐Goldberg et al. 2023; Asher 1992; Banerjee et al. 2017). Social support plays a pivotal role in buffering these negative effects; however, such support systems often diminish in the presence of addiction (Gavriel‐Fried et al. 2021).

This study aims to examine the effects of having an addicted spouse on marital communication and satisfaction among women in Turkey. The Family Peace Scale (FPS) was selected as the primary outcome measure because it comprehensively assesses marital satisfaction, family cohesion, and emotional well‐being, which are directly affected by partner addiction. The majority of existing literature has been conducted in Western countries, and due to cultural and social contextual differences, it does not directly reflect the experiences of women in Turkey. Therefore, there is a need for an original study that takes into account Turkey‐specific socio‐cultural factors. Additionally, the study seeks to provide insights for the development of effective family support mechanisms.

## 2 Methods

A consecutive sampling method was employed, including all eligible women who consented to participate. An a priori power analysis was conducted using G*Power 3.1 to determine the required sample size. Based on an expected effect size of Cohen's *d* = 0.5, *α* = 0.05, and power = 0.95, a minimum of 79 participants per group was indicated. During the study period, all eligible women who presented to the hospital and consented to participate were included, resulting in a total of 309 participants. The spouses of the women were addicted and hospitalized, while the women themselves were not addicted (women married to addicted men: 104; women married to non‐addicted men: 205). Prior to the acquisition of informed consent, each participant was furnished with pertinent information regarding the nature of the study.

A specific room was designated at the medical facility for female visitors to their male companions. The women were invited to participate in the study by completing a questionnaire if they were willing to do so. No identity information was collected during the data collection process. The women were invited to review the questionnaires that they had completed themselves, together with the researcher R.A. The researcher responsible for overseeing the data collection process was immediately available to ensure that the questionnaires had been completed correctly. Any missing questionnaires and incomplete answers were highlighted by the participants and requested to be amended. In accordance with hospital policy, the spouses of patients were included in the study following the provision of brief information to them.

The case group comprised married women aged 18 years and older, with the ability to read and write in Turkish, who were admitted to the hospital due to the addiction of their spouses to alcohol, drugs, or smoking. The control group comprised married women aged 18 years and over who were literate in Turkish, whose husbands did not have any addiction (alcohol, drugs, and smoking), and who applied to the outpatient clinic in the internal department of the hospital. The study excluded women who had any psychiatric disease, were unable to speak Turkish, and declined to participate. The women in the case and control groups who participated in the study exhibited comparable socio‐demographic characteristics, including age, educational status, and income.

The study form included the Introductory Information Form and FPS questionnaires, which included demographic information of the participant's questions about their addiction status and marital relationships.

**The descriptive information form** consists of a total of 20 questions developed by the researcher to determine the socio‐demographic information of women and their husbands, the addiction status of women and their husbands, and marital satisfaction.

**The Family Peace Scale (FPS)** is a 5‐point Likert‐type scale developed by Ozdemir and Bakiler (2021). The scale includes two sub‐dimensions (Deep Fraying and General Contentment) and 15 items, and it gives a total score. The eight items in the Deep Attrition sub‐dimension of the scale are scored in reverse  (eighth, ninth, tenth, eleventh, twelfth, thirteenth, fourteenth, and fifteenth). The higher the scores obtained from the scale, the higher the family peace. The Cronbach's alpha value is between 0.65 and 0.82. In the present study, the Cronbach's alpha value for the FPS was determined to be 0.96.

The data were analyzed using the Statistical Package for Social Sciences (SPSS) version 21. In descriptive statistics, the mean, standard deviation, minimum, and maximum values are provided for numerical variables, while the number and percentage values are provided for categorical variables. A *t*‐test was conducted to determine whether a significant discrepancy existed between the two groups. The analysis of variance (ANOVA) was employed to examine the differences between three or more groups. In case of a difference, the group(s) exhibiting a discrepancy were subjected to a pairwise comparison test. A *p*‐value of less than 0.05 was deemed to be statistically significant. Logistic regression analysis was conducted to examine the relationships between the dependent variable (man's addiction status) and independent variables; potential confounding factors (woman's educational level and economic status) were controlled in the analysis. In our study, the FPS exhibited a normal distribution across the variables, as assessed through the Skewness–Kurtosis test. Additionally, the homogeneity of the variances was evaluated with the Levene test, which confirmed the homogeneity of the variances.

## 3 Results

The mean age of the participants was 35.57 ± 9.58 years. It was observed that 38.2% of the participants were 37 years of age or older (*n*: 112), 52.4% of them had income equal to their expenses (*n*: 162), 47.9% had been married for 0–5 years (*n*: 148), 67.0% had at least a bachelor's degree (*n*: 207), and 64.4% of them had a spouse with at least a bachelor's degree (*n*: 199) (Table 1).

**TABLE 1:** The relationship between some descriptive characteristics of participants and FPS and sub‐factor totals (*n* = 309).

Characteristics	Number/ Percentage		FPS Total score	FPS Subfactor 1	FPS Subfactor 2
	*n*	%	$\bar{X}\pm SD$	$\bar{X}\pm SD$	$\bar{X}\pm SD$
**Age**					
18–30	109	35.3	53.88 ± 15.89	26.45 ± 07.21	27.43 ± 09.58
31–36	82	26.5	51.15 ± 15.00	25.79 ± 06.49	25.35±09.34
37 and above	112	38.2	53.49±13.93	26.34 ± 06.41	27.15 ± 08.56
*F*			0.886	0.247	1.373
*p*			0.414	0.781	0.255
**Economic condition**					
Income less than expenditure	68	22.0	49.87 ± 16.17	25.04 ± 07.40	24.82 ± 09.85
Income equals expenses	162	52.4	54.66 ± 14.63	26.94 ± 06.70	27.71 ± 08.84
Income exceeds expenses	79	25.6	53.01 ± 14.09	25.80 ± 05.93	26.52 ± 08.98
*F*			2.610	2.164	2.457
*p*			0.075	0.117	0.087
**Marriage duration**					
0–5 years	148	47.9	54.16 ± 15.43	26.49 ± 06.85	27.67 ± 09.36
6–10 years	69	22.3	52.67 ± 12.89	26.71 ± 05.65	25.96 ± 08.35
11–15 years	35	11.3	51.02 ± 16.71	24.91 ± 07.44	26.11 ± 09.95
≥ 15	57	18.4	51.63 ± 14.86	26.23 ± 06.70	25.82 ± 09.02
F			0.672	0.713	0.927
p			0.570	0.545	0.428
**Education status**					
≤ High school	102	33.0	48.08 ± 13.62	24.27 ± 06.81	23.80 ± 08.22
≥ Undergraduate	207	67.0	55.44 ± 14.97	27.20 ± 06.45	28.24 ± 09.25
*t*			−4.182	−3.677	−4.106
*p*			**<0.001**	**<0.001**	**<0.001**
**Spouse education status**					
≤ High school	110	35.6	48.85 ± 14.31	24.38 ± 06.97	24.46 ± 08.76
≥ Undergraduate	199	64.4	55.31 ± 14.79	27.26 ± 06.34	28.05 ± 09.13
*t*			−3.719	−3.681	−3.354
*p*			**<0.001**	**<0.001**	**<0.001**

A comparison of the descriptive characteristics of the participants with the mean scores of the FPS and its sub‐factors revealed that the difference between the mean scores for age, economic status, and duration of marriage was not statistically significant (*p* > 0.05). A statistically significant difference was observed between the mean scores of the Family Peace Scale (FPS) and its sub‐factors and the educational status of the spouse (*p* < 0.001, Table 1).

It was observed that 33.7% (n: 104) of the female participants had husbands who received addiction treatment, 61.2% (n: 189) had husbands addicted to any substance, 55.0% (n: 170) had husbands addicted to cigarettes, 15.2% (n: 47) had husbands addicted to alcohol, 28.5% (n: 88) had husbands addicted to drugs, and 4.9% (n: 15) had husbands addicted to medication (Table 2).

**TABLE 2:** The relationship between participant women's spouses' addictive characteristics and FPS and sub‐factor totals (*n* = 309).

Characteristics	Number/ Percentage		FPS Total score	FPS Subfactor 1	FPS Subfactor 2
	*n*	%	$\bar{X}\pm SD$	$\bar{X}\pm SD$	$\bar{X}\pm SD$
**Spouse's addiction treatment status**					
Spouse receiving addiction treatment	104	33.7	45.72 ± 11.53	23.06 ± 06.05	22.66 ± 07.00
Spouse not receiving addiction treatment	205	66.3	56.70 ± 15.11	27.84 ± 06.45	28.86 ± 09.42
*t*			−6.509	−6.290	−5.927
*P*			**<0.001**	**<0.001**	**<0.001**
**Any addiction of the spouse**					
No	120	38.8	59.11 ± 13.84	28.86 ± 05.89	30.25 ± 08.66
Yes	189	61.2	49.13 ± 14.32	24.57 ± 06.67	24.57 ± 08.78
*t*			6.048	5.764	5.576
*P*			**<0.001**	**<0.001**	**<0.001**
**Spouse addicted to smoking** ^*^					
No	139	45.0	57.17 ± 14.42	28.03 ± 06.22	29.14 ± 08.90
Yes	170	55.0	49.61 ± 14.50	24.76 ± 06.75	24.84 ± 08.92
*t*			4.570	4.381	4.215
*p*			**<0.001**	**<0.001**	**<0.001**
**Spouse addicted to alcohol** ^*^					
No	262	84.8	54.59 ± 14.84	26.91 ± 06.60	27.68 ± 09.14
Yes	47	15.2	44.17 ± 12.15	22.43 ± 06.02	21.75 ± 07.51
*t*			4.546	4.349	4.201
*p*			**<0.001**	**<0.001**	**<0.001**
**Drug addict spouse** ^*^					
No	221	71.5	56.39 ± 15.06	27.62 ± 06.59	28.77 ± 09.25
Yes	88	28.5	44.52 ± 10.66	22.76 ± 05.69	21.76 ± 06.67
t			6.745	6.068	6.466
p			**<0.001**	**<0.001**	**<0.001**
**Medication addict spouse** ^*^					
No	294	95.1	53.61 ± 14.77	26.51 ± 06.55	27.09 ± 09.11
Yes	15	4.9	41.27 ± 13.31	20.73 ± 07.50	20.53 ± 07.86
*t*			3.169	3.309	2.736
*p*			**0.002**	**0.001**	**0.007**

One person marked more than one answer.

A comparison of the mean scores on the Family Peace Scale (FPS) and its sub‐factors with the addiction characteristics of the spouses of the participants revealed a statistically significant difference between the mean scores of those whose spouses received addiction treatment, those whose spouses were addicted, those whose spouses were addicted to cigarettes, those whose spouses were addicted to alcohol, those whose spouses were addicted to drugs, and those whose spouses were addicted to medication (*p* < 0.01, Table 2).

**FIGURE 1:**
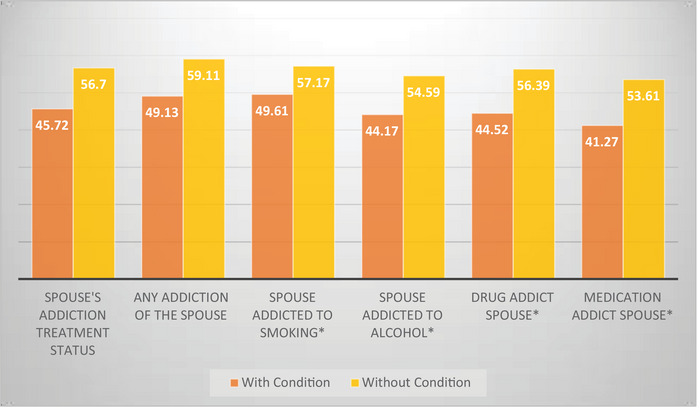
Distribution of FPS means according to spouse's addiction status.

As illustrated in Figure 1, women whose spouses had received addiction treatment exhibited lower Family Peace Scale (FPS) mean scores compared to those whose spouses had not. A similar pattern was observed across all categories of spousal addiction—including smoking, alcohol, drug, and medication dependence—whereby participants with addicted spouses consistently demonstrated lower FPS mean scores. These results suggest a negative association between spousal addiction and women's FPS outcomes (Figure 1).

According to the results of the logistic regression analysis, a higher educational level of the woman was associated with a reduced likelihood of the man being addicted (OR = 2.388; 95% CI: 2.166–2.907; *p* = 0.002). Conversely, the presence of any substance addiction in the woman significantly increased the odds of the man being addicted (OR = 2.422; 95% CI: 1.209–4.853; *p* = 0.013). In addition, lower Family Peace Scale (FPS) scores were associated with increased odds of the man being addicted (OR = 2.042; 95% CI: 2.021–2.064; *p* < 0.001), and the presence of sexual problems in the woman further elevated this likelihood (OR = 1.484; 95% CI: 1.270–1.868; *p* = 0.015) (Table 3).

**TABLE 3:** Results of logistic regression analysis regarding women's spouses' addiction to any substance.

	B	SE	Sig.	Exp(B)	95% C.I. for EXP(B)	
					Lower	Upper
**Economic status (Reference: Low)**			0.712			
**Economic status (Medium)**	0.051	0.385	0.894	1.053	0.495	2.241
**Economic status (High)**	−0.195	0.319	0.540	0.823	0.440	1.537
**Education status of women (Coveriate)**	−0.946	0.433	**0.002**	2.388	2.166	2.907
**Spouse education status**	0.534	0.398	0.179	1.706	0.783	3.720
**Marriage duration**	0.000	0.000	**0.001**	1.000	1.000	1.000
**Addiction status of the woman**	0.885	0.355	**0.013**	2.022	1.209	4.853
**Having sexual problems**	0.725	0.298	**0.015**	1.484	1.270	1.868
**FPS score**	−0.041	0.011	**<0.001**	2.042	2.021	2.064

Abbreviations: B: Regression coefficient, SE: Standard error, Wald: Chi‐square value, df: degrees of freedom, Sig.: Significance level (*p* < 0.05), Exp (B): Odds ratio (OR).

In Table 4, the marital satisfaction characteristics of the participant women whose husbands received and did not receive addiction treatment were compared. According to the table, a statistically significant difference was observed between those who received addiction treatment and those who did not, except for the questions “*I have more fun with my friends than with my husband*. and *I spend much time with my husband for games and entertainment*.” (*p* < 0.05, Table 4).

**TABLE 4:** Comparison of marital satisfaction characteristics of participant women whose husbands received and did not receive addiction treatment (*n* = 309).

Marriage satisfaction questions	Receiving addiction treatment		Not receiving addiction treatment		Total		Statistics
	*n*	%	*n*	%	*n*	%	
**Our relationship gives me happiness**.							
Yes	51	23.9	162	76.1	213	100	*X^2^*: 28.970 ***p*<0.001**
No	53	55.2	43	44.8	96	100	
**I have always been pleased with our relationship**.							
Yes	46	25.0	138	75.0	184	100	*X^2^*: 15.274 ***p*<0.001**
No	58	46.4	67	53.6	125	100	
**I have more fun with my friends than with my** spouse.							
Yes	44	40.0	66	60.0	110	100	*X^2^*: 3.078 *p*: 0.102
No	60	30.2	139	69.8	199	100	
**There is something wrong with our sexual relationship**.							
Yes	52	43.0	69	57.0	121	100	*X^2^*: 7.734 ***p*: 0.007**
No	52	27.7	136	72.3	188	100	
**I think my spouse is very attractive**.							
Yes	49	25.3	145	74.7	194	100	*X^2^*:16.469 ***p*<0.001**
No	55	47.8	60	52.2	115	100	
**If my spouse's family had not interfered with us so much, we could have had a happier relationship**.							
Yes	55	41.4	78	58.6	133	100	*X^2^*: 6.194 ***p*: 0.015**
No	49	27.8	127	72.2	176	100	
**When my spouse gets angry, he throws things around or throws them on the floor**.							
Yes	36	47.4	40	52.6	76	100	*X^2^*: 8.486 ***p*: 0.005**
No	68	29.2	165	70.8	233	100	
**My spouse and I spend a lot of time playing games and doing entertainment**.							
Yes	34	28.3	86	71.7	120	100	*X^2^*: 2.490 *p*: 0.138
No	70	37.0	119	63.0	189	100	
**There is an intense love and affection in our relationship**.							
Yes	43	24.2	135	75.8	178	100	*X^2^*: 16.969 ***p*<0.001**
No	61	46.6	70	53.4	131	100	
**We are not close enough to each other**.							
Yes	53	46.5	61	53.5	114	100	*X^2^*: 13.326 ***p*<0.001**
No	51	26.2	144	73.8	195	100	
**My spouse and I set the rules for the children together**.							
Yes	46	24.7	140	75.3	186	100	*X^2^*: 16.672 ***p*<0.001**
No	58	47.2	65	52.8	123	100	

*X^2^*: Chi‐square.

## 4 Discussion

The present study examined the relationship between women's demographic characteristics and family peace in the context of spousal addiction. Women whose husbands were undergoing addiction treatment or were addicted to any substance exhibited significantly lower FPS scores. Logistic regression analysis showed that a higher educational level of the woman was associated with a reduced likelihood of the man being addicted, whereas female substance addiction, higher FPS scores, and the presence of sexual problems were associated with an increased likelihood. These findings underscore the significant impact of partner behavior on marital and family dynamics. The findings of this study offer valuable insights for family counselors and social workers. In light of these results, further research is warranted to gain a more detailed understanding of the influence of demographic factors on family relationships.

The findings of this study indicate that there is a significant difference between the educational level of individuals and that of their spouses, as well as between the educational level of individuals and that of their families, with regard to the FPS and its sub‐factors. This provides an important indicator for understanding the dynamics of marital relationships. In the existing literature, there is a common emphasis on the decisive role that an individual's educational level plays in determining their quality of life, psychological well‐being, and social relationships (Antoniadou et al. 2023; Umberson and Montez 2010). While education provides individuals with problem‐solving abilities, it also enhances their communication and empathy skills (Defar et al. 2023). This may facilitate a more constructive approach to managing marital conflicts. Individuals with higher levels of education are more likely to have access to better economic opportunities and social support networks (Alegría et al. 2018). This may serve to mitigate marital stressors and reinforce communication within the family unit. Moreover, individuals with higher levels of education may possess greater knowledge and resources to cope with challenges such as addiction, which could potentially enhance marital satisfaction (Bradshaw et al. 2015; Mirzakhani et al. 2020). Marital satisfaction and family harmony are closely linked; positive relationship dynamics support overall family well‐being, while addiction can disrupt these dynamics (Randall and Bodenmann 2017). It is frequently reported in the literature that addiction has a deleterious effect on marital relationships (Bradshaw et al. 2015; Mirzakhani et al. 2020). While individuals struggling with addiction often create emotional and financial challenges for their partners, inevitably, their spouses will also experience negative effects as a result of this situation. A higher level of education may facilitate the development of more effective strategies for the management of addiction. For example, a recent study conducted in Turkey revealed that self‐esteem and educational level were positively correlated with life satisfaction and that increased life satisfaction was negatively correlated with addiction (Koccak et al. 2021). In this regard, it can be posited that an individual's educational status has the potential to mitigate the impact of addiction and that education is an effective factor in family dynamics. Accordingly, healthcare professionals and family counselors could develop programs aimed at enhancing awareness and coping skills among individuals with lower educational levels, while policymakers might implement social policies that expand educational opportunities and strengthen family resilience.

The findings of this study indicate that the level of education attained by women can exert an influence on the dynamics of the family unit and the addiction status of spouses. From the perspective of Family Systems Theory, this relationship can be better understood by recognizing that families function as interconnected systems—each member's behavior affects the others, and addiction in one individual can disrupt the overall family balance (Fals‐Stewart et al. 2004). In this context, women with higher education levels, who often possess stronger social support networks and more advanced communication skills, may be better equipped to maintain family stability and cope with addiction‐related challenges (Umberson and Montez 2010). This may assist them in more effectively coping with their husbands' addiction‐related issues. The findings of this study indicate that there is a significant relationship between women's educational status and men's addiction status. Furthermore, the results demonstrate that women play a pivotal role in determining the success of their partners in addiction treatment. It seems plausible to suggest that this is related to women having better social support networks and improved communication skills. It has been demonstrated that an elevated level of education is associated with enhanced stress‐coping abilities in women (Medical News Today 2023). This may also have an indirect effect on men's ability to cope with addiction problems. These findings suggest that the effective communication and adaptability skills developed by highly educated women (the Flexibility and Communication dimensions in Olson's Circumplex Model) support the family's ability to cope with addiction‐related stress and its role in maintaining general system balance (homeostasis) (Olson 2011; Olson and Defrain 2002). In this context, an increase in the educational level of women may have a beneficial impact on the management of men's addiction conditions.

Sexual problems have been demonstrated to exert a considerable influence on an individual's status with regard to addiction. The extant literature indicates that individuals experiencing sexual difficulties are at a higher risk of developing addictive behaviors (Bass and Nagy 2023; Brem et al. 2017). Sexual health problems are frequently linked to psychological and emotional challenges, which may elevate the likelihood of developing an addiction. In particular, it has been posited that those experiencing sexual dysfunction may resort to addictive behaviors as a means of coping with stress (Hartman et al. 2012; Goslar et al. 2020). The findings of this study indicate a correlation between sexual problems and addiction, which may have a detrimental impact on familial harmony. Addiction may result in difficulties within the context of sexual relationships. In particular, alcohol and substance addiction is associated with problems such as sexual reluctance and sexual dysfunctions (Goslar et al. 2020). Such issues have the potential to erode the quality of individuals' relationships with their partners, thereby contributing to familial unrest (Rokach and Chan 2023). As emphasized in Olson's Circumplex Model, the weakening of emotional closeness between spouses as a result of such problems may diminish family cohesion and push the family system toward unbalanced extremes (Olson 2011; Olson and Defrain 2002). Furthermore, the presence of sexual problems within a family unit may disrupt patterns of communication and emotional connectednes**s**, thereby deepening the overall sense of familial unrest (Rokach and Chan 2023). It can be, therefore, surmised that addiction may give rise to conflict among family members. For instance, an increased prevalence of sexual assault and violence has been observed among individuals with alcohol addiction, which may have a detrimental impact on familial dynamics (Huecker et al. 2023). These findings align with existing literature, suggesting that healthcare providers and family counselors could integrate sexual health evaluations into addiction treatment to enhance family harmony and reduce relapse risk.

It can be observed that marital satisfaction is an individual phenomenon and is directly correlated with the expectations held by the individual in relation to their spouse (Li et al. 2024). The term “family peace” is typically understood to signify the state of collective happiness and harmony experienced by the family unit as a whole. These findings demonstrate that individual satisfaction within the context of marriage can influence the level of family peace (Singh et al. 2023). The results of this study indicate that women who responded positively to marital satisfaction exhibited higher family peace scores, suggesting that emotional satisfaction is associated with an increase in peace within the family. The data indicated that women whose husbands had addiction problems exhibited lower levels of peace. As stated in the 2024 report from the Harmony Ridge Recovery Center, addiction is a complex problem that negatively affects not only the individual but also all family dynamics (Harmony Ridge Recovery Center 2024). These findings demonstrate a robust correlation between marital satisfaction and family peace. A healthy marriage and family environment are crucial factors influencing individual happiness. Conversely, addiction can negatively impact these dynamics, disrupting peace within the family (Singh et al. 2023; Harmony Ridge Recovery Center 2024). According to the Stress–Strain–Coping–Support (SSCS) model, the addictive behavior of one spouse functions as a chronic stressor within the family system, generating emotional strain among both partners and other family members. Such strain may lead to the adoption of maladaptive coping mechanisms, ultimately eroding marital communication, emotional intimacy, and overall family harmony (Orford et al. 2010). In this context, when the cumulative effects of stress and maladaptive coping diminish partners’ ability to communicate effectively and sustain emotional closeness, declines in marital satisfaction and disruptions in family peace become almost inevitable. The findings of this study not only support the theoretical predictions of the SSCS model but also show, empirically, that one partner's addiction directly negatively affects the other partner's emotional well‐being and family harmony, highlighting the need for targeted interventions to support affected families.

In this study, the prolongation of marital duration emerged as a factor associated with a reduced likelihood of addiction among men. This finding suggests that a stable and healthy marital environment may serve as a protective factor against the development of addictive behaviors. Furthermore, it underscores the potential effectiveness of marriage counseling, family support interventions, and educational programs in mitigating addiction‐related risks. Future research is warranted to further elucidate the mechanisms through which marital duration contributes to lowering the probability of addiction. The dynamics of a relationship and the level of commitment involved can vary considerably, even in the context of long‐term marriages. This can be attributed to a number of different factors. For example, one study reported that communication problems, conflicts, or external factors affect the level of commitment in marriage (Nemati et al. 2020). It is, therefore, important to address each relationship on its own merits rather than making generalizations about the relationship between marital duration and commitment (Stanley et al. 2010). In this context, the prolongation of the duration of marriage emerges as a factor that reduces the addiction status of men. However, it is essential to consider that each relationship should be evaluated within its own dynamics. It can thus be concluded that while a healthy marital environment may serve to reduce the risk of addiction, it is also evident that the distinctive dynamics of each relationship must be taken into account. This demonstrates the effectiveness of marriage counseling and support services, as well as marriage education, in fighting addiction. Furthermore, we posit that additional research is required to ascertain the impact of marital duration on the likelihood of addiction. These findings highlight that healthcare providers, family counselors, and policymakers could implement targeted interventions aimed at supporting long‐term marital stability, including the expansion of marriage education and counseling programs, as a practical strategy to mitigate addiction‐related risks within families.

Several limitations of this study should be acknowledged. First, the cross‐sectional design precludes the establishment of causal inferences regarding the observed relationships. Second, reliance on self‐reported measures may introduce response bias and social desirability effects. Third, the hospital‐based sample limits the generalizability of the findings to the broader female population. Furthermore, the exclusion of cultural and socio‐economic diversity may have overlooked the potential influence of these factors on family dynamics and addiction‐related outcomes.

## 5 Conclusion

This study shows that addictive behaviors harm marital satisfaction and family stability, whereas women's educational attainment and marital duration serve as protective factors that enhance family harmony and marital stability; accordingly, integrating family therapy and communication‐focused interventions into addiction treatment programs is recommended, and future research should utilize longitudinal designs and intervention‐based trials to examine causal mechanisms and assess the effectiveness of these interventions across diverse populations.

## Author Contributions

**Mehmet Ali Sen**: conceptualization, study design, formal analysis, and writing – original draft. **Eda Yakit Ak**: methodology, data visualization, writing – review and editing. **Ozden Tandogan**: data curation, validation, and investigation. **Ramazan Alkan**: data curation and investigation. **Ezgi Yarasir**: supervision, writing – review and editing.

## Funding

The authors have nothing to report.

## Ethics Statement

Ethics committee approval was obtained from the local institutional review board (Decision No: E‐14679147‐663.05‐710969).

## Consent

Informed consent form was obtained from the participants.

## Conflicts of Interest

The authors declare no conflicts of interest.

## Data Availability

Data will be shared with individuals upon request.
